# Recognition of Tumor Nidogen-1 by Neutrophil C-Type Lectin Receptors

**DOI:** 10.3390/biomedicines10040908

**Published:** 2022-04-15

**Authors:** Ronit Vogt Sionov, Chrystelle Lamagna, Zvi Granot

**Affiliations:** 1 Department of Developmental Biology and Cancer Research, Institute for Medical Research Israel Canada, Hadassah Medical School, The Hebrew University, Jerusalem 9112102, Israel; ronit.sionov@mail.huji.ac.il; 2 Rigel Pharmaceuticals, Inc., 1180 Veterans Boulevard, South San Francisco, CA 94080, USA; chrystelle.lamagna@gmail.com

**Keywords:** cancer, C-type lectin receptors, neutrophils, nidogen-1

## Abstract

Neutrophil-mediated cytotoxicity toward tumor cells requires cell contact and is mediated by hydrogen peroxide. We have recently shown that Cathepsin G expressed on the neutrophil surface interacts with tumor RAGE, and this interaction facilitates neutrophil cytotoxicity. Interruption of the Cathepsin G–RAGE interaction led to 50–80% reduction in cytotoxicity, suggesting that additional interactions are also involved. Here we show that blocking antibodies to the C-type lectin receptors (CLRs) Clec4e and Dectin-1, but not those to NKG2D, attenuated murine neutrophil cytotoxicity towards murine tumor cells, suggesting a contributing role for these CLRs in neutrophil recognition of tumor cells. We further observed that the CLRs interact with tumor Nidogen-1 and Hspg2, two sulfated glycoproteins of the basement membrane. Both Nidogen-1 and Hspg2 were found to be expressed on the tumor cell surface. The knockdown of Nidogen-1, but not that of Hspg2, led to reduced susceptibility of the tumor cells to neutrophil cytotoxicity. Altogether, this study suggests a role for CLR–Nidogen-1 interaction in the recognition of tumor cells by neutrophils, and this interaction facilitates neutrophil-mediated killing of the tumor cells.

## 1. Introduction

Neutrophils have versatile functions, being important for eliminating foreign invaders and in alerting the immune system when a danger is encountered [1]. In addition, they play important roles in tumor biology, where they exert both pro- and anti-tumor activities [2,3,4,5,6,7,8]. Besides secreting a whole battery of cytokines, chemokines and extracellular matrix (ECM)-modulating enzymes in response to activating stimuli, they also form cell–cell contact with endothelial and epithelial cells, which is important for their subsequent transendothelial and transepithelial migration [9,10]. In the context of cancer, interaction between neutrophils and tumor cells might either facilitate metastasis or lead to their elimination [2,3,4,7]. The outcome depends on the signals that neutrophils receive from the tumor cells and the tumor microenvironment, the activating status of the interacting neutrophils, and the susceptibility of the tumor cells to the lethal hit delivered by neutrophils. Whilst it is well-known that neutrophil killing of tumor cells involves hydrogen peroxide [11], the mechanisms involved in neutrophil recognition of tumor cells are still poorly understood. There are some reports that have attributed a pro-metastatic role for the interaction of neutrophil CD11b/CD18 (Mac-1) with ICAM-1 on selected tumor cells [12,13], and the interaction of neutrophil L-Selectin with mucin and non-mucin ligands on other tumor cell types [14,15]. In these cases, liver and lung neutrophils capture circulating tumor cells and promote their adherence to endothelial cells, a process required for their subsequent extravasation. We excluded an essential involvement for these interactions in facilitating the anti-tumor function of neutrophils (unpublished data). Rather, we observed an important role for the interaction of neutrophil surface expressed Cathepsin G with tumor RAGE in promoting neutrophil-mediated cytotoxicity towards the tumor cells [16].

Albeit the Cathepsin G–RAGE interaction is dominant in neutrophil cytotoxicity toward many different kinds of tumor cells, we believe that other interactions may also contribute to neutrophil tumor recognition. We therefore set forth to look for the involvement of some C-type lectin receptors (CLRs) on neutrophils. The reasoning behind this is the frequent aberrant glycosylation of tumor surface molecules [17], which are potential putative ligands for these lectin receptors. The CLR Mincle (Macrophage inducible C-type lectin, also termed Clec4e) has been shown to recognize yeast β-glucans [18], the glycolipid trehalose dimycolate of *Mycobacterium turberculosis* [19], and glycan residues on *Schistosome* eggs [20]. In addition, Clec4e recognizes endogenous ligands released from necrotic cells such as spliceosome-associated protein 130 (SAP130) [21], and as such, may sense damaged cells that should be expediently eliminated [22]. Dectin-1 (dendritic-cell-associated C-type lectin 1, also termed Clec7a) is another CLR that recognizes yeast β-glucans and is important for anti-fungal activity [23,24]. Dectin-1 has also been shown to facilitate the binding of the bacteria *Haemophilus influenza* to eosinophils [25] and is important for production of inflammatory mediators by macrophages in response to mycobacteria [26]. In addition, Dectin-1 interacts with the filament vimentin, leading to induction of superoxide anion production in monocytes [27].

In the present study, we identified a role for Clec4e and Dectin-1 on murine neutrophils in facilitating the anti-tumor response. We observed that soluble decoy receptors to the CLRs NKG2D, Clec4e and Dectin-1 partly interfered with neutrophil cytotoxicity towards murine tumor cells, suggesting a common ligand on tumor cells. Blocking antibodies to Clec4e or Dectin-1, but not those to NKG2D, interfered with the killing. We further show that Clec4e interacts with Dectin-1, suggesting that these receptors may act together. Inhibition of the CLR-associated SYK kinase didn’t interrupt neutrophil cytotoxicity, suggesting that the Clec4e/Dectin-1 receptor pair is important for recognition rather than delivering an outside–in signal through SYK. Importantly, we found that the CLRs interact with Nidogen-1 and Hspg2, two highly sulfated extracellular matrix glycoproteins that form part of the basement membrane. Both Nidogen-1 and Hspg2 were found to be expressed on the tumor cell surface. Knockdown of Nidogen-1, but not of Hspg2, in tumor cells reduced their susceptibility to neutrophil cytotoxicity, suggesting that Clec4e/Dectin-1 contribute to the recognition of tumor cells through interaction with Nidogen-1. Notably, the Nidogen-1/Hspg2 complexes also interact with tumor RAGE, suggesting that these extracellular matrix molecules strengthen the neutrophil-tumor cell synapse by bridging CLRs with RAGE.

## 2. Materials and Methods

### 2.1. Mice

5–6-week-old BALB/c and C57BL/6 mice were purchased from Harlan (Israel). In vivo tumor growth was done by injecting 0.5 × 10^5^ 4T1 or AT3 tumor cells in 50 μL PBS into the mammary fat pad. Primary tumor growth was measured with a digital caliper and tumor volume calculated by the formula 0.52 × width^2^ × length. Control and Fostamatinib (R788; SYK inhibitor) containing diet was kindly provided by Rigel Inc., South San Francisco, CA, USA. All experiments involving animals were approved by the Hebrew University's Institutional Animal Care and Use Committee (IACUC).

### 2.2. Neutrophil Isolation

Mouse neutrophils were purified from 8–12-week-old BALB/c mice that have been injected orthotopically with 1 × 10^6^ 4T1 cells, or 8–12-week-old C57BL/6 mice that have been injected orthotopically with 5 × 10^5^ AT3. Neutrophil purification was done as previously described [28]. The SYK inhibitor R408 was kindly provided by Rigel Inc., South San Francisco, CA, USA.

### 2.3. Cell Culture

Mouse 4T1 breast cancer cells and mouse Lewis lung carcinoma cells (LLC) were purchased from ATCC and cultured in DMEM containing 7.5% heat-inactivated FCS (Sigma, St. Louis, MI, USA). Mouse AT3 PyMT breast cancer cells were kindly provided by Prof. Scott Abrams, Roswell Park Cancer Institute (Buffalo, NY, USA) and cultured in DMEM containing 7.5% heat-inactivated FCS. The cell cultures were tested to be mycoplasma-free using the EZ-PCR™ Mycoplasma Test Kit (Biological Industries, Kibbutz Beit-HaEmek, Israel).

The tumor cells were transduced with a retroviral vector (MigR1-Luc) to stably express firefly luciferase. For soluble receptor expression, cells were infected with viral particles prepared from tet-inducible pLV_TRE_RFP vector (kindly provided by Prof. Eli Keshet, The Hebrew University of Jerusalem, Israel) expressing the extracellular part of the respective receptors, and mRFP-positive cells were sorted using BD FACSARIA III cell sorter. Soluble receptor expression was induced by adding 1 μg/mL doxycycline (Sigma) to the cells the day before assaying. Nidogen-1 and Hspg-2 knockdown cells were prepared by lentiviral transduction with either Nidogen-1 specific shRNAs (TRCN0000114787 and TRCN0000114790; Sigma) or Hspg2 specific shRNAs (TRCN0000246980 and TRCN0000246981; Sigma) followed by puromycin selection (2 μg/mL for 5 days).

### 2.4. Plasmid Preparations

Soluble C-type lectin receptors were prepared by cloning the extracellular part of the receptor fused downstream to human IL-2 secretion signaling peptide, separated by either Flag-His or Fc fragment, and inserted into the tet-inducible pLV_TRE_RFP plasmid kindly provided by Prof. Eli Keshet (The Hebrew University of Jerusalem, Israel). The human IL-2 secretion signaling peptide was prepared by primer dimerization of 5′-aat tcg ccg cca cca tgt aca gga tgc aac tcc tgt ctt gca ttg cac taa gtc ttg cac ttg tca caa aca gta-3′/5′-cgc gta ctg ttt gtg aca agt gca aga ctt agt gca atg caa gac agg agt tgc atc ctg tac atg gtg gcg gcg-3′ forming an EcoRI site at the 5′ and a MluI site at the 3′. 6xHis-Flagx3 tag was prepared by mixing the two primer pairs: 5′-cgc gtc atc atc acc atc acc atg gtg act aca agg acc atg acg gtg-3′/5′-ttg taa tca ccg tca tgg tcc ttg tag tca cca tgg tga tgg tga tga tga-3′ and 5′ att aca agg atc atg aca tcg act aca agg atg acg atg aca agg gta-3′/5′-ccg gta ccc ttg tca tcg tca tcc ttg tag tcg atg tca tga tcc-3′ providing an MluI site at the 5′ and AgeI at the 3′. The Fc fragment was prepared by amplifying the Fc fragment of the CSI-Ig (Fc mut)-IRES-puro plasmid kindly provided by Prof. Ofer Mandelboim (The Hebrew University of Jerusalem) using Phusion Flash High-Fidelity PCR master mix and the primer pair: 5′-cag tac gac gcg tga acc caa gag ctg cga caa g-3′/5′-cag tac gac cgg tct tgc cag ggg aca ggc tca ggc tc-3′, and cut with MluI/AgeI. The mutant Fc fragment of human IgG1 does not bind Fc receptors, and as such will not encourage antibody-dependent cell-mediated cytotoxicity [29]. Extracellular part of mouse NKG2D (NM_033078.4) was prepared by amplifying bone marrow cDNA using the primer pair: 5′-cag tac gac cgg ttt caa aga gac gtt tca gcc ag-3′ (AgeI site and nt 378–399)/5′-gat cct agc tag ctt aca ccg ccc ttt tca tgc ag-3′ (NheI site and nt 809–788 with stop signal). Extracellular part of mouse Clec4e (NM_019948.2) was prepared by amplifying neutrophil cDNA using the following primer pairs: 5′-cag tac gac cgg tac ata tcg cag ctc tca aat ttc cgg g-3′ (AgeI site and nt 259–285)/5′-gat cct agc tag ctt agt cca gag gac tta ttt ctg gca tgt g-3′ (NheI site and nt 768–742 with a stop signal). Extracellular part of mouse Dectin-1 (NM_020008.2) was prepared by amplifying neutrophil cDNA using the following primer pairs: 5′-cag tac gac cgg tcg aca caa ttc agg gag aaa tcc ag-3′ (AgeI site and nt 303–327)/5′-aag gct act agc tag ctt aca gtt cct tct cac aga tac tgt atg-3′ (NheI site and nt 827–799 with a stop signal). sRAGE-Fc fusion protein was prepared as described [16] encompassing nt 45–1049 of RAGE transcript variant 1, NM_007425.3. sE-Cadherin-Fc was prepared by exchanging sRAGE with sE-Cadherin. The latter (NM_009864.3) was prepared by amplifying cDNA from embryonic stem cells using the primer pair: 5′-act tcc gga att cgc cgc cac cat ggg agc ccg gtg ccg cag ctt ttc cg-3′ (EcoRI site and nt 146–173)/5′-cag tac gac cgg taa ctt gca atc ctg ctg cca cg-3′ (AgeI site and nt 2272–2251). Nidogen-1 (NM_010917.2) was prepared by amplifying cDNA from AT3 cells using the primer pair: 5′-cag tac gac cgg tgc cgc cac cat gat gct gga cgc gag cgg ctg tag-3′ (AgeI site and nt 101–123)/5′-aag gct act agc tag ctt tcc gtt caa tgc agt caa c-3′ (NheI site and nt 3835–3815). Flagx3 was prepared by annealing the primer pair: 5′-cta gcg act aca agg acc atg acg gtg att aca agg atc atg aca tcg act aca agg atg acg atg aca agt gag c-3′/5′-ggc cgc tca ctt gtc atc gtc atc ctt gta gtc gat gtc atg atc ctt gta atc acc gtc atg gtc ctt gta gtc g-3′ that have overhanging NheI and NotI sites and a stop codon. The Fc was prepared as above, but using the NheI/NotI restriction sites. The Nidogen-1 link area (aa 270–356) was amplified using the primer pairs: 5′-cag tac gac cgg tgc cgc cac cat gac cgc caa ggg cgt ggt gtc tgc-3′ (AgeI site and nt 908–930)/5′-aag gct act agc tag cct ccg ctg gca gct gga aag atc-3′ (NheI site and nt 1168–1146) and cloned into the pLV_TRE_RFP harboring a Flagx3 tag. The Nidogen-1 G2 region (aa 357–665) was amplified using the primer pairs: 5′-cag tac gac cgg tgc cgc cac cat gag gtt ccc tca gca tca ccc cca gg-3′ (AgeI site and nt 1169–1193)/5′-aag gct act agc tag cgg cat cag ggg agc cat ccc tca c-3′ (NheI site and nt 2095–2072).

### 2.5. Antibodies

The following primary antibodies were used for Western blot analysis: Mouse anti-Flag (Sigma, Clone M2), Rat anti-Nidogen (Santa Cruz Biotechnology, Santa Cruz, Bolivia, Clone ELM1), anti-Heparan sulfate glycoprotein (Perlecan) (Merck, Darmstadt, Germany, MAB1948P; Clone A7L6) and Biotin-SP-AffiniPure Goat anti-human Fcγ fragment (Jackson ImmunoResearch Laboratories Inc., Ellsworth, ME, USA, 109-065-098). HRP-conjugated secondary antibodies against the respective species and HRP-conjugated streptavidin were purchased from Jackson ImmunoResearch Laboratories Inc. The following neutralization antibodies were used: Rat anti-mouse Mincle (InvivoGen, San Diego, CA, USA, Clone 6G5), Rat anti-mouse Dectin (InvivoGen; Clone R1-8g7), Armenian hamster anti-mouse NKG2D Clone C7 (kindly provided by Prof. Ofer Mandelboim, the Hebrew University of Jerusalem), LEAF^TM^ purified Rat anti-mouse NKG2D (Biolegend; San Diego, CA, USA, Clone CX5). The following antibodies were used for flow cytometry: Rabbit anti-Clec4e (Novus Biologicals, Littleton, CO, USA, NB110-62089), Alexa Fluor^647^-conjugated Rat anti-mouse Dectin 1 (Clone 2A11; Bio-Rad, Hercules, CA, USA); and APC-conjugated anti-mouse NKG2D (CD314) (Miltenyi Biotec, Koln, Germany). APC-conjugated AffiniPure F(ab)_2_-Fragment Donkey anti-Human Fcγ fragment (Jackson ImmunoResearch Laboratories Inc., 709-136098) was used to detect Fc-fusion proteins on flow cytometry.

### 2.6. PCR Primer Pairs

The following RT-PCR primer pairs were used: Clec4e (Mincle) (NM_019948.2): 5′-atg aat tca acc aaa tcg cct gc-3′/5′-tta gtc cag agg act tat ttc tgg cat c-3′; DAP10 (Hcst) (NM_011827.3): 5′-ccc agg cta cct cct gtt c-3′/5′-cta caa tta gga gtg aca tga ccg-3′; DAP12 (tyrobp) (NM_011662.2) 5′-ctg gga ttg ttc tgg gtg ac-3′/R: 5′-ctg aag ctc ctg ata agg cg-3′; Dectin-1 (Clec7a) (NM_020008.2): 5′-atg aaa tat cac tct cat ata gag-3′/5′-tta cag ttc ctt ctc aca gat act gta tg-3′; R: HPRT (NM_013556.2): 5′-gtt ctt tgc tga cct gct gga t-3′/5′-aac ttt tat gtc ccc cgt tga ct-3′; Hspg2 (Perlecan) (NM_008305.3): 5′-gcc tgc cct gtt tct gca t-3′/5′-gca cca gaa gtt cat cca gat ct-3′; NKG2D (KLRK1) (NM_033078.4): 5′-acg ttt cag cca gta ttg tgc-3′/5′-gga agc ttg gct ctg gtt c-3′; Nidogen-1 (Entactin) (NM_010917.2): 5′-ttc cgc tgc gag tgt gta ga-3′/5′-caa gga tgt gct ctc gtt cca-3′. For real-time PCR the following primer pairs were used: Nidogen-1 (Entactin) (NM_010917.2): 5′-ccg atg cct tct gct aca aca-3′/5′-gat gtg ctc tcg ttc cag ttg a-3′; Hspg2 (Perlecan) (NM_008305.3): 5′-ccg cct ctc ttt cag caa ct-3′/5′-gag agg gtg tac cgc agc tt-3′; TBP (NM_013684.3): 5′-ccg tga atc ttg gct gta aac tt-3′/5′-cag ttg tcc gtg gct ctc tta tt-3′.

### 2.7. Neutrophil Cytotoxicity Assay

Luciferase-containing mouse tumor cells (10,000 cells per 96-flat-bottomed white well, Corning) were plated in 100 μL OptiMEM containing 2% heat-inactivated FCS 18–20 h before adding neutrophils. The following day, purified mouse normal high-density neutrophils (100,000 cells per well) were added in 50 μL OptiMEM containing 2% heat-inactivated FCS to the plated tumor cells and co-cultured overnight. Luciferase activity was measured using a Tecan F200 microplate reader as described [28]. D-Luciferin free acid was purchased from AnaSpec Inc. (Fremont, CA, USA). (82,250) and ATP from Sigma (St. Louis, MI, USA). The percentage of tumor cell killing was calculated by the following formula: (1 − Luc_Neut_/Luc_Cont_) × 100, where Luc_Neut_ is the luciferase activity in tumor cells following neutrophil interaction and Luc_Cont_ the luciferase activity in control tumor cells.

For blocking experiments, neutralizing antibodies were pre-incubated with neutrophils for 30 min prior to their addition to the tumor cells. Various concentrations of the antibodies were used (100 ng/mL to 1 μg/mL). For the induction of soluble receptors, the tumor cells were incubated in the presence of 1 μg/mL doxycycline (Sigma).

### 2.8. Co-Immunoprecipitation

Control or Fc-fusion protein with or without Flag-tagged protein-expressing tumor cells were lysed in 50 mM Tris HCl pH 8.0, 300 mM NaCl, 1 mM MgCl_2_, 10 mM KCl, 0.5% NP-40, 10% glycerol containing EDTA-free protein inhibitor cocktail (Sigma), and incubated with protein A-Sepharose beads (Sigma) for 2 h. The beads were washed 4 times in the lysis buffer before analysis of co-immunoprecipitated proteins. MS/MS mass spectrometry of the samples was performed at the Smoler Protein Research Center at the Technion University, Haifa. Proteome Discoverer version 1.4 was used for peptide identifications. All data sets were searched with Mascot and SEQUEST (with probability score calculation). Peptides detected in Protein A-precipitated Fc-fusion protein samples are presented in Table 1.

### 2.9. Statistical Analysis

For studies comparing differences between two groups, we used unpaired Student′s *t* tests. Differences were considered significant when *p* < 0.05. Data are presented as average ± SEM of 3–5 experiments, unless otherwise stated.

## 3. Results

### 3.1. Soluble Decoy C-Type Lectin Receptors Limit Neutrophil Cytotoxicity

**Figure 1:**
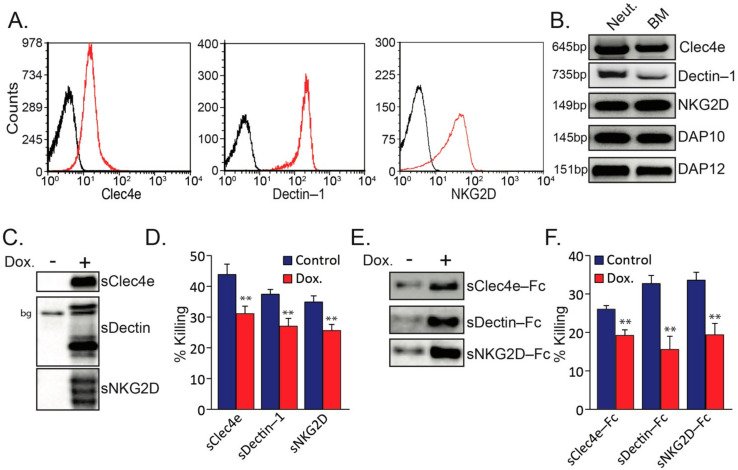
(**A**) Surface staining of tumor-elicited high-density neutrophils for Clec4e, Dectin-1 and NKG2D. (**B**) RT-PCR of indicted genes. Neut. = Neutrophils; BM = Bone marrow. (**C**) WB of supernatants from AT3 overexpressing tet-inducible form of Flag-tagged soluble sClec4e, sDectin and sNKG2D in the absence (Control) or presence of 1 μg/mL doxycycline (Dox.). bg = background band. (**D**) The sensitivity of AT3 overexpressing tet-inducible form of soluble sClec4e-Flag, sDectin-Flag and sNKG2D-Flag to neutrophil cytotoxicity. (**E**) WB of supernatant from AT3 overexpressing tet-inducible form of soluble sClec4e-Fc, sDectin-1-Fc and sNKG2D-Fc in the absence (Control) or presence of 1 μg/mL doxycycline (Dox.). (**F**) The sensitivity of AT3 overexpressing tet-inducible form of soluble sClec4e-Fc, sDectin-Fc and sNKG2D-Fc to neutrophil cytotoxicity. ** *p* < 0.001.

Neutrophils express several C-type lectin receptors (CLRs) that are potential receptors for recognition of aberrant glycosylation on tumor cells [30]. In addition to the well-documented expression of Clec4e and Dectin-1 on neutrophils ([30]; Figure 1A,B), we noticed that neutrophils also express the NKG2D receptor (Figure 1A,B), which was previously reported to play a role in NK recognition of tumor cells [31,32]. In addition, the neutrophils express the two adaptor proteins DAP10 and DAP12 (Figure 1B) which are known to be associated with NKG2D [33] as well as with other immune cell receptors [34]. To test whether these C-type lectin receptors play a role in mediating neutrophil cytotoxicity, we overexpressed a soluble form of the extracellular domains of NKG2D, Clec4e and Dectin-1 (Figure 1C) that act as decoy molecules. We observed that soluble receptors prepared from all three CLRs consistently inhibited neutrophil-mediated cytotoxicity towards AT3 tumor cells by 25–30% (Figure 1D). Similarly, using a modified construct of the soluble receptors fused to a mutant Fc [29], all three fusion proteins interfered with the killing of AT3 cells by neutrophils (Figure 1E,F).

### 3.2. Blocking Antibodies to Clec4e and Dectin-1 Inhibit Neutrophil Tumor Cytotoxicity

**Figure 2:**
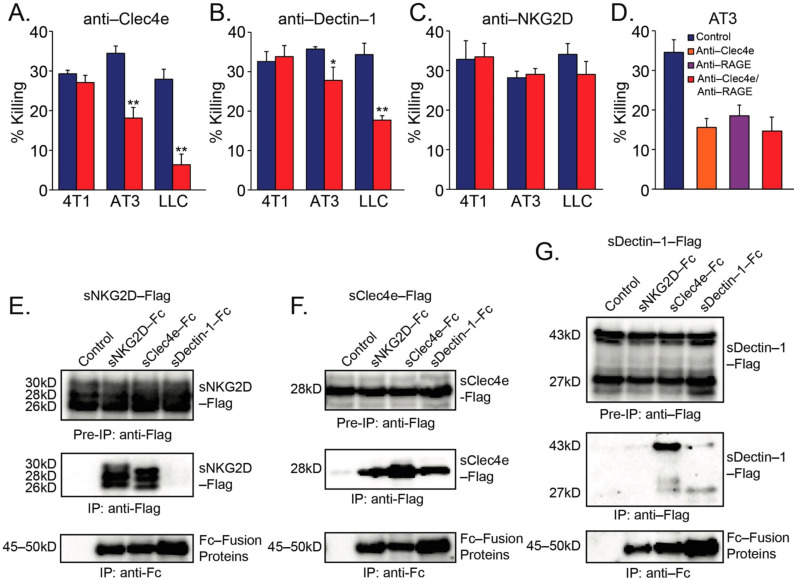
(**A**) Inhibition of neutrophil cytotoxicity using blocking antibodies to Clec4e. (**B**) Inhibition of neutrophil cytotoxicity using blocking antibodies to Dectin-1. (**C**) Blocking antibodies to NKG2D did not perturb neutrophil cytotoxicity. (**D**) The concomitant presence of both anti-Clec4e (1 μg/mL) and anti-RAGE (1 μg/mL) did not cause a stronger inhibition of neutrophil cytotoxicity towards AT3 cells than either antibody alone. (**E**–**G**) Immunoprecipitation of C-type lectin receptor Fc fusion proteins in the presence of sNKG2D-Flag (**E**) sClec4e-Flag (**F**) or sDectin-1-Flag (**G**) Pre-IP = Cell extract samples prior to immunoprecipitation. IP = Immunoprecipitated samples. * *p* < 0.05; ** *p* < 0.001.

To provide further support to the notion that CLRs mediate neutrophil recognition of tumor cells, we pre-incubated the neutrophils with blocking antibodies to Clec4e, Dectin-1 or NKG2D prior to their incubation with the tumor cells. Blocking antibodies to Clec4e or Dectin-1 significantly inhibited the neutrophil-mediated killing of AT3 and LLC cells (Figure 2A,B), whereas antibodies targeting NKG2D had no significant effect (Figure 2C). Notably, none of these antibodies affected neutrophil-mediated killing of 4T1 breast cancer cells (Figure 2A–C), which are apparently recognized by another, yet unknown, mechanism. Since tumor RAGE is important for the recognition of tumor cells by neutrophils [16], we wondered whether CLR recognition of tumor cells is an independent event or acts in concert with RAGE. Combining anti-Clec4e with anti-RAGE had no additive effect on neutrophil cytotoxicity, suggesting that these molecules could be part of the same recognition mechanism (Figure 2D).

The finding that antibodies to both Clec4e and Dectin-1 reduced tumor cell killing, led us to test whether Clec4e and Dectin-1 could interact with each other, and thus act together. To this end, we overexpressed various combinations of Fc- or Flag-fused CLRs in AT3 cells and tested which CLRs co-immunoprecipitate. We observed that NKG2D and Clec4e, but not Dectin-1 could homodimerize (Figure 2E–G). Importantly, Clec4e could interact with both NKG2D and Dectin-1, while no interactions between NKG2D and Dectin-1 could be observed (Figure 2E–G). These observations, together with the inhibitory effects of antibodies to Clec4e or Dectin-1, suggest that Clec4e and Dectin-1 may co-operate in recognizing the tumor cells.

### 3.3. SYK Activity Is Not Required for the Anti-Tumor Function of Neutrophils

**Figure 3:**
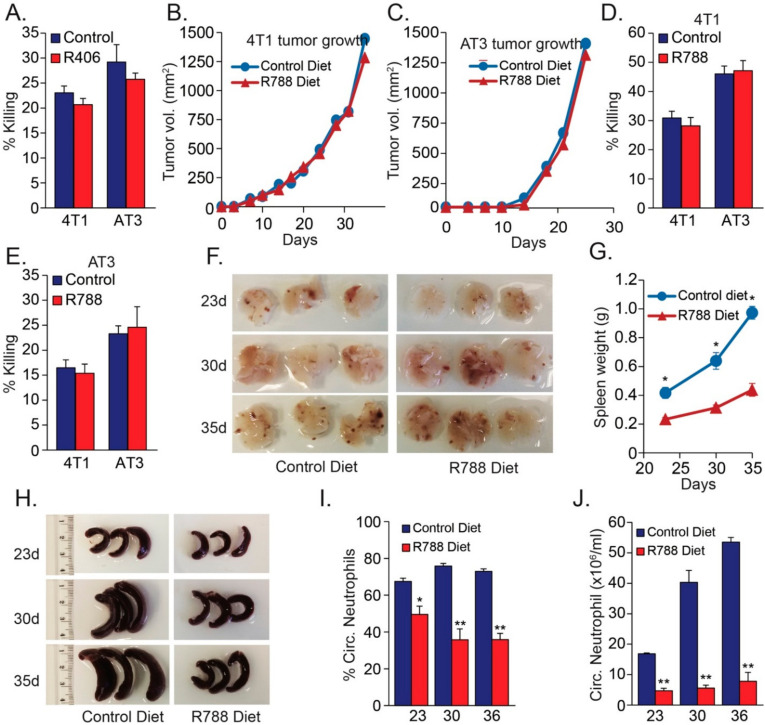
(**A**) Cytotoxicity of neutrophils pretreated in vitro 30 min with 10 μM SYK inhibitor R406 towards 4T1 and AT3 cells. (**A**–**C**) Local tumor growth of 4T1 (**B**) or AT3 (**C**) in mice that have been given control or R788-containing diet. (**D**,**E**). Cytotoxicity of neutrophils isolated from 4T1 (**D**) or AT3 (**E**)-tumor bearing mice that have been given control or R788-containing diet. (**F**) Lungs from 4T1-bearing mice that have been given control or R788-containing diet for 23, 30 and 35 days. (**G**,**H**) Spleen from 4T1-bearing mice that have been given control or R788-containing diet for 23, 30 and 35 days. (**I**) Percentage neutrophils in blood samples from 4T1-bearing mice that have been given control or R788-containing diet for 23, 30 and 35 days. (**J**) The neutrophil count in 1 mL blood samples from 4T1-bearing mice that have been given control or R788-containing diet for 23, 30 and 35 days. * *p* < 0.05; ** *p* < 0.001.

CLRs are known to interact with the immunoreceptor tyrosine-based activation motif (ITAM)-containing adaptor molecule Fc receptor γ-chain (FcRγ) that becomes phosphorylated upon ligand binding. This in turn leads to the recruitment of spleen tyrosine kinase (SYK) and the activation of downstream signaling pathways important for immune cell activation [22,35,36]. Based on these facts, we raised the question whether the involvement of CLRs in anti-tumor neutrophil activity is due to outside-in signaling that affects neutrophil activation. To test this possibility, we treated neutrophils with the SYK inhibitor R406 (10 μM) prior to their addition to the tumor cell culture. Both control and R406-treated neutrophils killed tumor cells to a similar extent (Figure 3A), suggesting that SYK-dependent signaling is dispensable for this particular neutrophil function. To further study the role of SYK signaling, mice that have been injected orthotopically with either 4T1 or AT3 tumor cells, were given either a SYK inhibitor (R788, Fostamatinib)-containing diet or a control diet. There was no significant difference in tumor growth when comparing control and R788-treated mice (Figure 3B,C). Neutrophils isolated from R788-treated mice showed similar cytotoxicity towards tumor cells as those isolated from control mice (Figure 3D,E), excluding a central role for SYK signaling in the generation of anti-tumor neutrophils. Similar numbers of lung metastases were observed in R788-treated and control mice (Figure 3F). Of note, R788 strongly prevented tumor-induced splenomegaly (Figure 3G,H) and neutrophilia (Figure 3I,J). Thus, the SYK inhibitor affects neutrophil production without disturbing their anti-tumor function.

### 3.4. Interaction of C-Type Lectin Receptors with Nidogen-1

**Figure 4:**
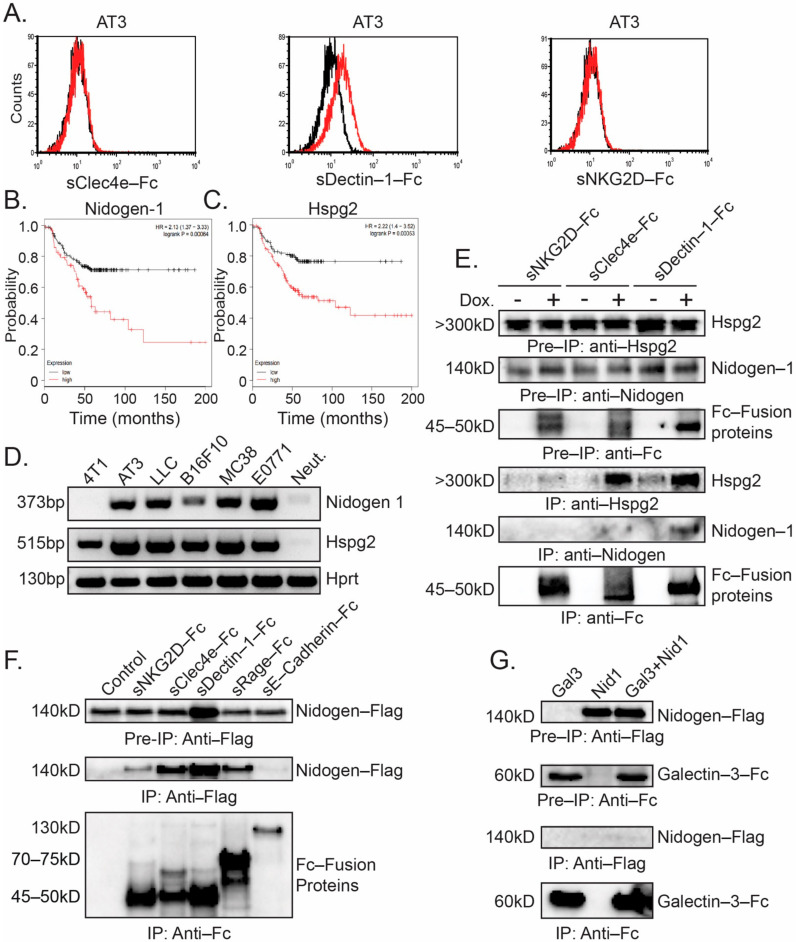
(**A**) Binding of sDectin-Fc to the cell surface of AT3 cells. AT3 cells were incubated with supernatant containing sClec4e-Fc, sDectin-1-Fc or sNKG2D-Fc, followed by incubation with APC-anti-Fc antibodies (red histograms). Black histograms represent 2nd antibody alone. (**B**) Kaplan-Meier Plot of survival of Her-2 positive breast cancer patients with low (black) or high (red) expression of Nidogen-1. (**C**) Kaplan-Meier Plot of survival of Her-2 positive breast cancer patients with low (black) or high (red) expression of Hspg2. (**D**) RT-PCR analysis of Nidogen-1 and Hspg2 expression in various tumor cell lines and in neutrophils (Neut.). (**E**) Co-immunoprecipitation of endogenous Nidogen-1 and endogenous Hspg2 in AT3 cells overexpressing tet-inducible Fc fusion proteins as indicated. Induction of Fc fusion proteins was done by adding 1 μg/mL doxycycline (Dox.) to the culture. Pre-IP = Cell extract samples prior to immunoprecipitation. IP = Immunoprecipitated samples. (**F**) Immunoprecipitation of Nidogen-1-Flag using the indicated Fc fusion proteins as baits. The proteins were overexpressed in AT3 cells. (**G**) Immunoprecipitation study of Nidogen-1-Flag using Galectin-3-Fc fusion protein as a bait. The proteins were overexpressed in AT3 cells. Pre-IP = Cell extract samples prior to immunoprecipitation. IP = Immunoprecipitated samples.

We next wanted to study whether the Fc-CLR fusion proteins could bind to tumor cells. Surprisingly, significant binding was observed only by sDectin-Fc to AT3 tumor cells (Figure 4A). To identify the binding partners of CLRs, we immunoprecipitated extracts of control AT3 cells and AT3 cells overexpressing CLR-Fc fusion protein using protein A-beads. The co-immunoprecipitated proteins were analyzed by mass spectrometry (Table 1). We noted that extracellular matrix proteins of the basement membrane (Laminins, Heparan sulfate proteoglycan (Hspg; Perlecan) and Nidogen-1 (Entactin)) were common for all three receptors. Collagen-alpha-1 (I) chain co-immunoprecipitated with sNKG2D-Fc, but not with sClec4e-Fc or sDectin-1-Fc (Table 1). Among these ECM proteins, Nidogen-1 and Hspg2 were of particular interest, not only because they are sulfated glycoproteins [37,38], but also because their overexpression has been associated with a more malignant phenotype ([38,39,40] and Figure 4B,C). RT-PCR analysis shows that with the exception of 4T1 (lacking the expression of Nidogen-1), all cell lines express both Nidogen-1 and Hspg2 (Figure 4D). We next wanted to verify the MS data showing an interaction of C-type lectin receptors with Nidogen-1 and Hspg2. To this end, AT3 cells overexpressing CLRs fused to Fc were subjected to immunoprecipitation followed by Western blotting using antibodies to either Nidogen-1 or Hspg2. We observed a strong interaction of Clec4e and Dectin-1 but not NKG2D with endogenous Hspg2 and Nidogen-1 (Figure 4E). We further studied the CLR–Nidogen-1 interaction by overexpressing Nidogen-1-Flag and Fc-fusion proteins of sNKG2D, sClec4e and sDectin-1 in AT3 cells followed by immunoprecipitation. Indeed, we observed that sClec4e and sDectin-1 interacted with Nidogen-1-Flag, while a much weaker interaction was observed between sNKG2D and Nidogen-1-Flag (Figure 4F). In this assay, we also included sRAGE-Fc, which we recently showed to strongly reduce neutrophil cytotoxicity towards AT3 and LLC [16], and the non-related sE-Cadherin-Fc fusion protein (which has no effect on neutrophil cytotoxicity). Interestingly, sRAGE-Fc bound Nidogen-1-Flag to a certain extent (Figure 4F), suggesting that there may be a functional/physical link between tumor cell Nidogen-1 and RAGE and the C-type lectin receptors on neutrophils. No interaction could be observed between sE-Cadherin-Fc and Nidogen-1 (Figure 4F). Neither could any interaction between the Galectin-3-Fc fusion protein and Nidogen-1 be observed (Figure 4G) indicating for specific interactions between CLRs and Nidogen-1.

### 3.5. shRNA to Nidogen-1 Reduced Tumor Sensitivity to Neutrophil Cytotoxicity

**Figure 5:**
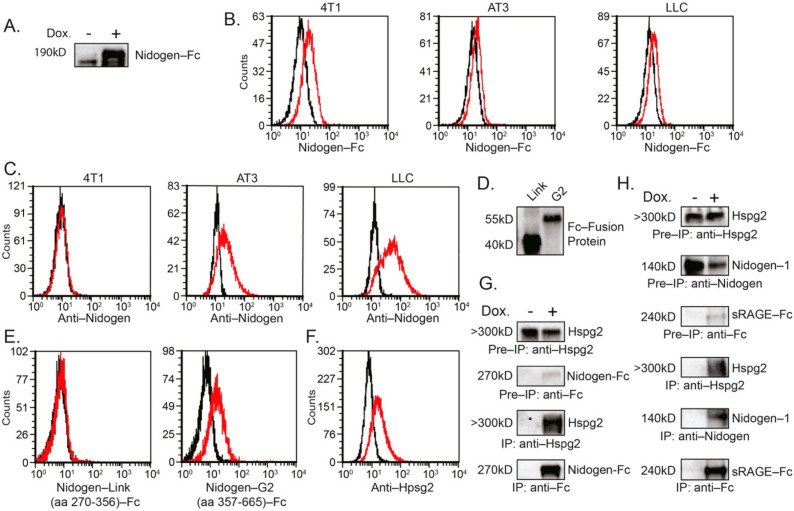
(**A**) Western blot analysis of Nidogen-Fc expression in supernatants of AT3 cells used for cell binding studies in (**B**). (**B**) Nidogen-1-Fc binding to tumor cells. The cells were incubated with supernatant containing Nidogen-1-Fc followed by incubation with APC-anti-Fc antibodies (red histograms). Black histograms represent cells incubated with control supernatant and APC-anti-Fc antibodies. (**C**) Staining of tumor cells with anti-Nidogen-1 antibodies followed by 2nd FITC-anti-rat antibodies (red histograms). Black histograms represent cells incubated with 2nd antibody only. (**D**) Western blot analysis of Nidogen link region (aa 270–356) fused to Fc and Nidogen G2 region (aa 357–665) fused to Fc. (**E**) Binding of Nidogen-G2 region-Fc fusion protein to AT3 cells. AT3 cells were incubated with supernatant containing either the Nidogen link region (aa 270–356) fused to Fc or Nidogen G2 region (aa 357–665) fused to Fc followed by incubation with APC-anti-Fc antibodies (red histograms). Black histograms represent cells incubated with control supernatant and APC-anti-Fc antibodies. (**F**) Staining of AT3 cells with anti-Hspg2 antibodies followed by 2nd FITC-anti-rat antibodies (red histograms). Black histograms represent cells incubated with 2nd antibody only. (**G**) Co-immunoprecipitation of endogenous Hspg2 in AT3 cells overexpressing tet-inducible Nidogen-1-Fc fusion protein. Induction of the Fc fusion protein was done by adding 1 μg/mL doxycycline (Dox.) to the culture. Pre-IP = Cell extract samples prior to immunoprecipitation. IP = Immunoprecipitated samples. (**H**) Co-immunoprecipitation of endogenous Nidogen-1 and endogenous Hspg2 in LLC cells overexpressing tet-inducible sRAGE-Fc fusion protein. Induction of Fc fusion proteins was done by adding 1 μg/mL doxycycline (Dox.) to the culture. Pre-IP = Cell extract samples prior to immunoprecipitation. IP = Immunoprecipitated samples.

Flow cytometry analysis using Nidogen-1-Fc fusion protein (Figure 5A) shows that Nidogen-Fc could bind to the cell surface of 4T1, AT3 and LLC cells (Figure 5B). Notably, Nidogen-1 is expressed on both AT3 and LLC cells but not on 4T1 cells (Figure 4D and Figure 5C). We further show that Nidogen-1 binds to the surface of tumor cells through the G2-domain (Figure 5D,E) which is known to be the binding domain of Nidogen-1 to Hspg2 [41]. Since Hspg2 is also expressed on the surface of the tumor cells (Figure 5F), and Nidogen-1 interacts with Hspg2 ([41] and Figure 5G), it is likely that Nidogen-1 binds to tumor cell surface through Hspg2. Interestingly, co-immunoprecipitation studies show that sRAGE-Fc binds endogenous Hspg2 and Nidogen-1 (Figure 5H), suggesting that tumor RAGE may contribute to their binding to the tumor cell surface.

**Figure 6:**
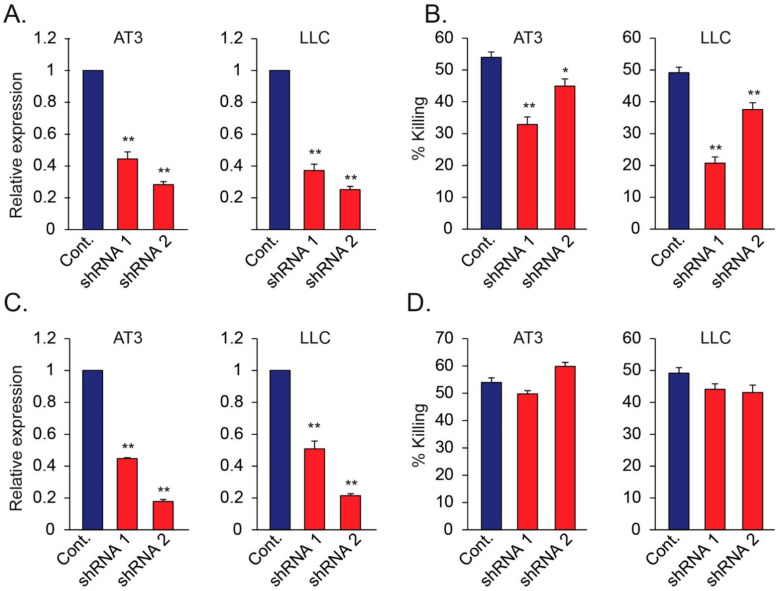
(**A**,**B**) Relative mRNA levels of Nidogen-1 in AT3 and LLC cells following transduction of two different shRNAs targeting Nidogen-1 (**A**), and the resulting susceptibility of these cells to neutrophil cytotoxicity (**B**). (**C**,**D**) Relative mRNA levels of Hspg2 in AT3 and LLC cells following transduction of two different shRNAs targeting Hspg2 (**C**), and the resulting susceptibility of these cells to neutrophil cytotoxicity (**D**). * *p <* 0.05; ** *p* < 0.001.

To study the involvement of tumor expressed Nidogen-1 in neutrophil cytotoxicity, we used specific shRNA to knock down the expression of Nidogen-1 and Hspg2 in AT3 and LLC cells (Figure 6A,B). Control, Nidogen-1 knocked down and Hspg2 knocked down cells were then cocultured with neutrophils. shRNA to Nidogen-1, but not shRNA to Hspg2, caused significant reduction in the sensitivity of tumor cells to neutrophil-mediated cytotoxicity (Figure 6C,D), suggesting a role for Nidogen-1 in neutrophil recognition of tumor cells.

**Figure 7:**
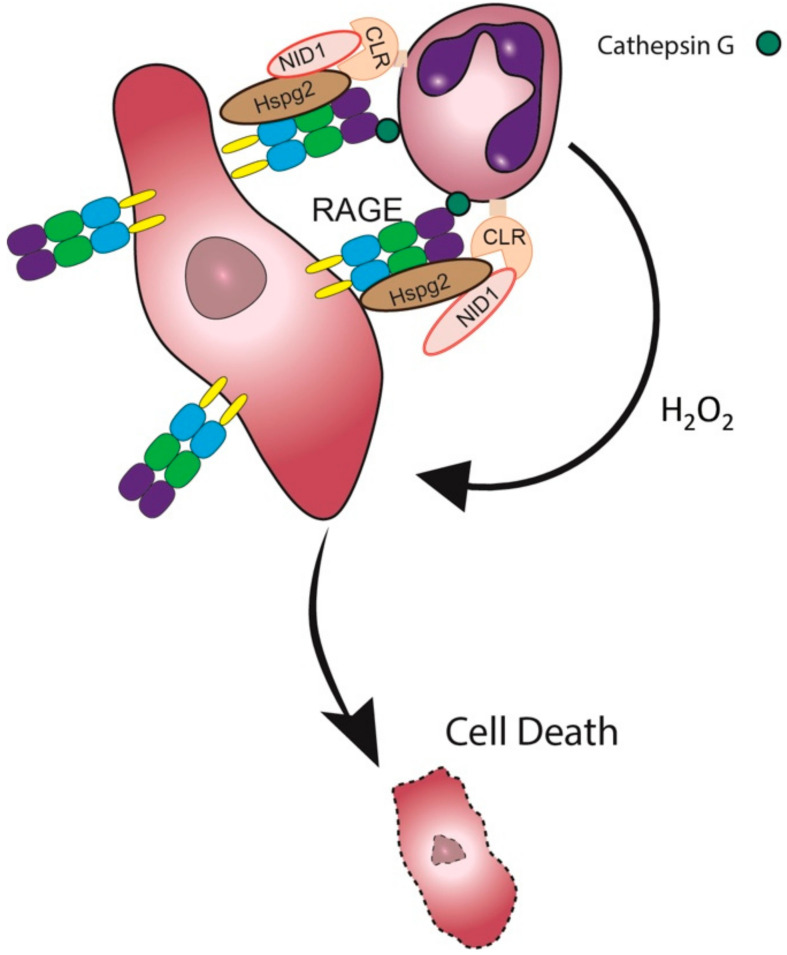
A proposed model of the neutrophil-tumor cell synapse. CLR – C-type Lectin Receptor; NID1 – Nidogen-1.

Taken together, our study suggests that CLR recognition of tumor Nidogen-1 may facilitate the neutrophil-tumor cell interaction required for the subsequent cytotoxic step (Figure 7).

Tumor cells expressing RAGE on their cell surface will be recognized by Cathepsin G expressed on the neutrophils [16]. In addition, the CLR receptors Dectin-1 and Clec4e on neutrophils interact with Nidogen-1/Hspg2 expressed on the tumor cell surface, some of which also interact with the tumor RAGE, thus bridging the neutrophils to the tumor cells. The neutrophils produce hydrogen peroxide into the synapse, which is the lethal hit that induces tumor cell death [11,42].

## 4. Discussion

Neutrophils have been known for decades to exert anti-tumor functions in virtue of their ability to interact with the tumor cells and to produce hydrogen peroxide [2,6,11,42]. To achieve the anti-tumor response, the neutrophils have to form direct cell contact with the tumor cells [11]. However, the mechanisms used by neutrophils to recognize tumor cells are only partly understood. We recently observed that Cathepsin G expressed on the neutrophil surface interacts with tumor RAGE, and this interaction is important for forming the neutrophil–tumor cell synapse required for tumor cell killing [16]. Importantly, Cathepsin G knockout neutrophils exhibited impaired tumor cytotoxicity toward RAGE-proficient tumor cells, and RAGE knockout tumor cells showed limited susceptibility to neutrophil cytotoxicity [16]. While the Cathepsin G–RAGE interaction is important for neutrophil cytotoxicity, we assumed that other interactions might also support the neutrophil–tumor cell synapse required for subsequent cell killing. We focused this study on C-type lectin receptors, since tumor cells frequently show aberrant glycosylation that might be recognized by these receptors. Specifically, we studied the involvement of the three C-type lectin receptors (CLRs) Clec4e, Dectin-1 and NKG2D. This report is the first one to describe NKG2D expression on neutrophils. Soluble forms of the three CLRs prepared by the extracellular part of the molecules, partly interfered with neutrophil cytotoxicity, suggesting for a common ligand and/or a co-operation between the receptors. The use of neutralizing antibodies demonstrated a role for Clec4e and Dectin-1, but excluded a central role for NKG2D. Further studies showed that Clec4e and Dectin-1 interact with each other, which might explain the inhibition of neutrophil anti-tumor function by using neutralizing antibodies to either Clec4e or Dectin-1. A similar co-operation has been observed between Dectin-2 and Dectin-3 on macrophages, where both receptors are required to mount an efficient anti-fungal response [43].

Since Clec4e and Dectin-1 are known to deliver outside–in signals through spleen tyrosine kinase SYK, we wondered whether SYK inhibition could interfere with neutrophil cytotoxicity. However, neutrophils treated with SYK inhibitor showed intact tumor cytotoxicity, excluding this possibility. Of note, the SYK inhibitor strongly prevented tumor-induced neutrophilia and splenomegaly in mice. We therefore assumed instead that the CLRs interact with a tumor component. The search for such a ligand was done by immunoprecipitation using Fc-fusion proteins of the CLRs as baits, followed by mass spectrometry. This approach revealed several components belonging to the basement membrane including Laminins, Nidogen-1, Heparan sulfate proteoglycans and Collagen-alpha-1 (I) chain. These components are known to interact with each other, so their presence in the co-immunoprecipitation could be either a direct interaction with the CLRs or through one of the components. We set forth to look at Nidogen-1 and Hspg2, as these are highly sulfated glycoproteins implicated in cancer metastases [38,39,40], and we (this paper) and others [44,45] observed that these basement membrane components are also expressed on the cell surface. We performed further studies to verify the mass spectrometry data, and observed that endogenous Nidogen-1 binds to various extents to the three CLRs studied, with the strongest interaction with Dectin-1, followed by Clec4e, and the least with NKG2D. Endogenous Hspg2 was also found to interact with Clec4e and Dectin-1, with a much weaker interaction with NKG2D. Moreover, Nidogen-1 was found to bind tumor cell-associated Hspg2, suggesting that these components form complexes on the cell surface. Interestingly, the Nidogen-1/Hspg2 complex was also found to interact with tumor RAGE. While tumor RAGE interacts strongly with Cathepsin G expressed on the neutrophil cell surface [16], we did not observe any interaction between the CLRs and Cathepsin G (unpublished data), suggesting that another receptor on neutrophils is responsible for the binding of Cathepsin G to neutrophils. The finding that both tumor RAGE and CLRs interact with Hspg2/Nidogen-1, suggests that Hspg2/Nidogen-1 might bridge between RAGE and CLRs, and thus strengthen the neutrophil-tumor cell synapse. The presence of RAGE and CLRs in the same synapse is further strengthened by the lack of synergy of neutralizing antibodies to RAGE and Clec4e.

Although Nidogen-1 is an essential component of the basement membrane, we observed that Nidogen-1 is also expressed on the tumor cell surface, and that the Nidogen-Fc fusion protein can bind to the tumor cells. This makes Nidogen-1 a tumor component that can be recognized by neutrophils. To study its involvement in neutrophil cytotoxicity, we used shRNA to knockdown Nidogen-1 or Hspg2. While shRNA to Hspg2 barely affected neutrophil cytotoxicity, shRNA to Nidogen-1 reduced tumor cell susceptibility to neutrophil cytotoxicity, suggesting that Nidogen-1 contributes to the neutrophil recognition of tumor cells required for the subsequent cytotoxic effect.

Our data are interesting in light of the fact that neutrophil function is dependent on their ability to transverse the endothelial cell monolayer and the basement membrane of the blood vessel endothelium and migrate into the interstitial extracellular matrix to reach the site of injury. Most studies have focused on the role of β-integrins in the interaction with components of the ECM such as Collagen, Fibronectin, Laminin and Vitronectin [46,47]. Our study adds the CLRs Clec4e and Dectin-1 to the list of receptors on neutrophils that can recognize ECM components. Besides Nidogen-1 and Hspg2 described here, Dectin-1 has been shown to interact with the filament vimentin [27]. A crosstalk between Dectin-1 and β-integrins has also been demonstrated [48]. Interestingly, Chiba et al. [49] observed that Dectin-1 expressed on dendritic cells and macrophages positively regulates the anti-tumor function of NK cells toward tumor cells that express high levels of N-glycan structures. They further observed that tumors grow faster in mice lacking the Dectin-1 protein, which is compatible with our data showing a role for Dectin-1 in the anti-tumor function of neutrophils. The observation that the NK activating CLR receptor NKp44 also interacts with both Nidogen-1 [44] and Heparan sulfate proteoglycans [50], suggests some shared features between NK cells and neutrophils. Adding on top of this the expression of the NK receptor NKG2D on neutrophils, suggests that neutrophils and NK cells are more closely related.

## 5. Conclusions

Using mouse models of cancer, we have presented data showing that the two CRLs Dectin-1 and Clec4e on neutrophils are co-operating in recognizing Nidogen-1 and Hspg2 on the tumor cell surface. RAGE is one of the receptors on the tumor cells that assist keeping Nidogen-1 and Hspg2 on the tumor cell membrane. Interruption of Nidogen-1 expression reduces the tumor cell susceptibility to neutrophil cytotoxicity, which accords with our previous data demonstrating that knockout of RAGE expression confers resistance to neutrophil cytotoxicity [16]. Taken together, Nidogen-1 on the tumor cell surface contributes to the interaction between neutrophils and tumor cells required for the subsequent tumor cell death.

## Figures and Tables

**Table 1:** Mass Spectrometry Data for tumor cell proteins interacting with Fc-sNKG2D, Fc-sClec4e and Fc-sDectin-1.

Accession	Protein	Σ Coverage	Σ# Protein	Σ# Unique Peptide	Σ# PSMs	Fc-sNKG2D (Area)	Fc-Clec4e (Area)	Fc-sDectin-1 (Area)
F8VQJ3	Laminin subunit gamma-1	10.89	3	14	88	1.925 × 10^7^	8.281 × 10^7^	3.603 × 10^7^
Q61292	Laminin subunit beta-2	7.28	2	11	48	3.451 × 10^6^	4.698 × 10^7^	1.810 × 10^7^
P02469	Laminin subunit beta-1	4.82	3	7	22	9.095 × 10^6^	1.997 × 10^7^	1.118 × 10^7^
E9PZ16	Basement membrane-specific Heparan sulfate proteoglycan core protein	1.44	3	4	16	1.046 × 10^6^	4.527 × 10^6^	5.450 × 10^6^
P10493	Nidogen-1	3.05	1	3	19	3.083 × 10^6^	1.988 × 10^7^	2.289 × 10^7^
B1AWE0	Clathrin light chain A	16.20	7	3	5	2.437 × 10^7^	4.102 × 10^6^	None
P18760	Cofilin-1	21.08	3	3	21	2.358 × 10^7^	5.776 × 10^7^	1.571 × 10^7^
P11087	Collagen alpha-1(I) chain	3.10	1	3	9	5.032 × 10^7^	None	None
A0A0G2JGD2	Protein S100-A4 (Fragment)	21.79	2	3	21	None	1.474 × 10^8^	None
P10852	4F2 cell-surface antigen heavy chain	6.84	3	3	8	None	8.170 × 10^6^	None
P35564	Calnexin	7.95	1	4	7	None	2.422 × 10^7^	None
O35639	Annexin A3	15.48	4	5	19	None	None	1.071 × 10^7^
Q64727	Vinculin	6.66	1	6	17	None	None	6.481 × 10^6^
P50543	Protein S100-A11	37.76	1	4	19	None	None	3.347 × 10^7^

Accession: The unique identifier assigned to the protein by the FASTA database. Σ Coverage: Displays by default the percentage of the protein sequence covered by identified peptides. Σ# Protein: Displays the number of identified proteins in the protein group of a master protein. Σ# Unique Peptide: The number of peptide sequences unique to the protein group. Σ# PSM: The total number of identified peptide sequences (peptide spectrum matches) for the protein. Area: The average area of the three unique peptides with the largest peak area.

## Data Availability

Not applicable.
